# Artificial fairness? Trust in algorithmic police decision-making

**DOI:** 10.1007/s11292-021-09484-9

**Published:** 2021-09-12

**Authors:** Zoë Hobson, Julia A. Yesberg, Ben Bradford, Jonathan Jackson

**Affiliations:** 1 Institute for Global City Policing, Department of Security and Crime Science, University College London, 35 Tavistock Square, WC1H 9EZ London, UK; 2 Department of Methodology, London School of Economics and Political Science, London, UK; 3 Sydney Law School, Sydney, Australia

**Keywords:** Algorithms, Fairness, Police decision-making, Technology, Trust

## Abstract

**Objectives:**

Test whether (1) people view a policing decision made by an algorithm as more or less trustworthy than when an officer makes the same decision; (2) people who are presented with a specific instance of algorithmic policing have greater or lesser support for the general use of algorithmic policing in general; and (3) people use trust as a heuristic through which to make sense of an unfamiliar technology like algorithmic policing.

**Methods:**

An online experiment tested whether different decision-making methods, outcomes and scenario types affect judgements about the appropriateness and fairness of decision-making and the general acceptability of police use of this particular technology.

**Results:**

People see a decision as less fair and less appropriate when an algorithm decides, compared to when an officer decides. Yet, perceptions of fairness and appropriateness were strong predictors of support for police use of algorithms, and being exposed to a successful use of an algorithm was linked, via trust in the decision made, to greater support for police use of algorithms.

**Conclusions:**

Making decisions solely based on algorithms might damage trust, and the more police rely solely on algorithmic decision-making, the less trusting people may be in decisions. However, mere exposure to the successful use of algorithms seems to enhance the general acceptability of this technology.

**Supplementary Information:**

The online version contains supplementary material available at 10.1007/s11292-021-09484-9.

## Introduction

The use of artificial intelligence and algorithmic decision-making now permeates many parts of society and the economy, with an increasing number of government agencies as well as private sector entities considering—and indeed using—this type of technology. In the summer of 2020, just a few months before the experiment described in this paper took place, the use of algorithms was suddenly thrown into the public eye in the UK with the announcement that, as a result of the COVID-19 pandemic, A-Level exam results in England had been calculated in this way. This resulted in serious implications for the students sitting the exams, with the “downgrading” of almost 40% of results (Coughlan, 2020). The resulting furore led to a withdrawal of the algorithmically determined grades and the use of teacher-predicted marks instead.

In the light of such developments—including, pertinent to the current paper, concerns about “algorithmic justice” (Huq, 2019)—a good deal of public and scholarly attention has recently focused on the use of algorithms to aid or replace human decision-making (Dhasarathy et al., 2020). This work has raised a number of questions concerning the fairness and consistency of algorithmic decisions. Like other public sector organizations, the police have begun incorporating new technologies into their working environment, with a range of programmes, trials and other implementations either in place now or currently under development. The use of algorithms and artificial intelligence by police—along with data analytics and the broader shift to “data-driven policing” (Kearns & Muir, 2019)—has been driven both by the reduction of resources for the public sector and the availability of digital technology (Ferguson, 2017; David and Ola, 2020). Due to their potential for high accuracy, low cost, effectiveness and efficiency, artificial intelligence and algorithmic tools can be appealing to public services (Shrestha & Yang, 2019). Use cases range from Live Facial Recognition technology to identify wanted people (Fussey & Murray, 2019), predictive policing (David and Ola, 2020), risk assessment (Oswald et al., 2018) and the Most Serious Violence Tool (Home Office, 2019). More widely, there is an expectation that police should have the same access and ability to utilise technology as the general public (Mackey, 2020), and private sector actors and police use of new technologies in many ways merely mirrors wider societal change.

It seems likely that more and more responsibility will be handed to these new technologies and that there will be greater reliance on policing decisions made primarily by machines (Ridgeway, 2019). However, while the potential benefits are notable, there is also scepticism and concern about the fairness of such systems, and their potential to inadvertently discriminate against, for example, certain minority groups. Previous research has highlighted concerns about potential negative effects arising from the use of predictive policing tools (Ferguson, 2017; Couchman, 2019; David and Ola, 2020), most notably in relation to the inability of the technology to take all relevant information into account, and the potential for “baking in” disproportionality and discrimination (Babuta & Oswald, 2020; Brayne, 2020).

In this paper, we explore how the use of algorithmic tools to make operational policing decisions affects lay reactions to police activity. Public reactions to the introduction of algorithmic decision-making in policing are comparatively understudied, but at the threshold it seems there are likely to be two inter-related sets of concerns in play. First, will people trust the decisions made by algorithms? Will they view policing decisions made by machines as more or less trustworthy than those made by human actors? As we describe below, the literature on public views of algorithmic decision-making suggests people can hold a complex and quite subtle set of opinions, and the answers to these and related questions remain unclear. Second, will people trust the police to use this technology appropriately? A growing body of research has explored public trust as a critical factor that shapes public attitudes and acceptance towards police uptake of new technology (see among many others Ariel et al., 2018; St Louis et al., 2019; Meijer & Wessels, 2019; Ridgeway, 2019; Bradford et al., 2020). Such trust is of course not “free-floating” or entirely prior to the development under consideration, but is developed via the direct, vicarious and mediated experiences people have of policing (Jackson et al., 2013). The current study uses an experimental approach to test public acceptability and support for (or opposition to) the use of algorithmic tools in the context of operational policing decisions, specifically, and support for (or opposition to) police use of this technology in a general sense.

## Who or what is making the decision, and does it have trustworthy motives?

We know (or think we know) how we make decisions and we have (or think we have) some insight into the potentially complex amalgamation of information that goes into the decision-making process. However, most people are much less aware of how machines can make these same calculations and decisions (Grzymek & Puntschuh, 2019; Lee, 2018). Evidence suggests, however, that algorithmic decision-making processes are perceived as having less agency and emotional capabilities than humans, therefore rendering algorithmic decision-makers more rational and less intentional or emotional (Lee, 2018). Statistical models are seen as more accurate than humans at predicting various outcomes across several disciplines (Kleinberg et al., 2018). Within healthcare, for instance, algorithmic tools are predicted to perform with expert-level accuracy and deliver cost-effective healthcare at scale—often outperforming human healthcare providers (Longoni et al., 2019). It is easy to imagine that this superior accuracy would be preferable to many, with people willing to follow the advice of the data-driven technology over human intuition.

Yet, research has suggested that more weight is often placed on advice given by a human expert compared to an algorithm (Dietvorst et al., 2015; Önkal et al., 2009); people are, for example, more likely to follow the recommendation of a physician than of a computer (Promberger & Baron, 2006). Indeed, Longoni and colleagues (2019) demonstrated, across a variety of medical decisions, a robust reluctance to use algorithms and artificial intelligence compared to human care providers. Averseness to healthcare delivered by artificial intelligence—to the prospect of being cared for by a decision-making machine—may evoke a concern that one’s unique characteristics, circumstances and symptoms will be neglected by a “cold”, impersonal machine that lacks motives that could be deemed trustworthy or untrustworthy.

The type of decision to be made (and/or what the decision is) also seems to influence public perceptions. Lee (2018) found that when the decision-maker (either algorithmic or human) was making a managerial decision about a mechanical task (for example scheduling employees’ shift patterns), algorithm and human-made decisions were perceived as equally fair and trustworthy. However, differences appeared when the decision-maker was considering a human task (for example whether to arrest someone, although that was not a focus of Lee’s study); here, algorithms were perceived as less fair and trustworthy and evoked more negative emotions than human decisions. People seem to think that algorithms lack intuition and subjective judgement capabilities (Lee, 2018). Applied to the current context, this ability to understand and incorporate unique characteristics, while showing sensitivity, could be key if the police were to adopt such technology and for it to be accepted by the public. Each situation the police are presented with will have its own specific qualities that need to be taken into consideration, and people may be reluctant to accept a machine can do this adequately.

The phrase “algorithmic aversion” (Dietvorst et al., 2015) has been used to describe why people might be wary of or opposed to the use of algorithmic decision-making in a context such as policing. Burton et al. (2018) set out five sets of reasons behind such aversion, including false expectations that affect responses to algorithmic decision-making (for example the idea that error is systematic, “baked in” and therefore irreparable); concerns about a lack of decision control and an emphasis on the need for human decision-making in contexts marked by uncertainty, where “alternatives, consequences, and probabilities are unknown and optimization is unfeasible” (ibid: 226)—i.e. where people feel there is not enough formalised information to make algorithmic decision-making a plausible option.

Relatedly, the importance of trust in generating acceptance of AI and related technologies has also been emphasised. Drawing on Mayer et al.’s (1995: 712) widely cited definition of trust, “the willingness of a party to be vulnerable to the actions of another party based on the expectation that the other will perform a particular action important to the trustor, irrespective of the ability to monitor or control that other party”, Glikson and Woolley, 2020: 629) argue that trust among users will predict the extent of reliance on a new technology and that this can take positive and negative forms: “Low trust in highly capable technology would lead to disuse and high costs in terms of lost time and work efficiency … whereas high trust in incapable technology would lead to over-trust and misuse, which in turn may cause … undesirable outcomes”. Based on a systematic review, they outline the characteristics of trustworthy AI, including tangibility or presence, reliability, transparency, immediacy (e.g. ability to respond to human presence and speech) and, under some conditions, anthropomorphism. AI technology that does not display these characteristics is less likely to be trusted and its use is thus less likely to be supported. Of particular note, given the vignettes used in the experiment described below, is that Glikson and Woolley (ibid) suggest that in the context of “embedded AI” where there is no visual representation or “identity”, reliability and transparency are likely to be particularly important factors.

## Public perceptions of police use of new technology

Traditional UK policing relies heavily on the Peelian ideology of policing by consent, in which public views of police legitimacy and trustworthiness are based on transparency about, and integrity in, the use of police powers, accountability and justice. On this account, while police should, and indeed must, embrace new technology, they also need to understand the ethical issues arising from doing so. There is also a strong need to test the acceptability of new technologies since implementing new tools that transgress boundaries of appropriateness (Huq et al., 2017; Trinkner et al., 2018) risks significant damage to public trust.

There are important issues of privacy, fairness and accountability involved when policing relies on algorithmic technology to inform decisions that can have an impact on the liberty of the individual or on the generation of outcomes they favour (Mackey, 2020). In the context of police decision-making, procedural justice has been found to be a key factor in generating public trust and police legitimacy (Mazerolle et al., 2013). Procedural justice relates to the fairness, consistency and accuracy of the decision-making process, and the quality of interpersonal interaction across dimensions of dignity, respect and voice, and it has been found to be central to generating support for decisions, sometimes irrespective of their favourability to the people involved (Brockner & Wiesenfeld, 2005; Brown et al., 2019), satisfaction with the decision-maker and outcome achieved (Tyler, 2006) and other outcomes including institutional trust, legitimacy and compliance (Tyler & Jackson, 2014). The literature on policing—rather unlike that on work organisations (e.g. Colquitt, 2001)—also regularly finds that procedural justice is more important than distributive justice (typically defined as the fair allocation of policing outcomes across aggregate social groups) in shaping people’s responses to authority (Hinds & Murphy, 2007; Reisig et al., 2007; Sunshine & Tyler, 2003; Tankebe, 2009; Tyler & Wakslak, 2004). One reason for this may be that, while in employment situations people can often see the outcomes provided to others (i.e. co-workers), this is less often the case in policing, where the wider outcomes achieved by police are often hidden from those involved in any one interaction. Indeed, this may lead people to infer distributive justice from procedural justice in policing contexts (Solomon & Chenane, 2021, c.f. van den Bos et al., 1997a, b) .

According to procedural justice theory, the effect or outcome of officer decision-making is therefore only one-factor driving public trust and support for police actions. Perceptions of the nature of the decision-making process and the quality of interpersonal treatment can be equally if not more important, and indeed, a “good” process can make up for a “bad” outcome. People tend to support police decisions they believe have been made in the right way, even if the outcome of those decisions is not favourable to them (Tyler, 2006). One important reason for this is that procedural justice generates a sense of motive-based trust in the decision-maker—that they are at least trying to do the right thing for the right reasons and have the interests of the trustor in mind—and this mitigates the effect of any failure to actually achieve desired ends (Tyler & Huo, 2002).

It might be imagined that procedural justice should be a prominent feature in algorithmic decision-making, and of people’s perceptions of it, as this technology follows the same set of rules and procedures every time. Furthermore, algorithms can sometimes be perceived as higher in quality and objectivity (Sundar & Nass, 2001) as they are not influenced by emotional factors or overt biases. All this should enhance trust. However, perceived trust is often lower for algorithmic rather than human decision-making because people do not believe that algorithms have the ability to learn from their mistakes (Dietvorst et al., 2015) or the capacity to successfully execute a task (Lee, 2018). Just the fact that decisions are made by algorithms rather than by humans may influence perceptions of the decisions that are made, regardless of the qualities of the decision outcomes (Lee, 2018).

Concerns about issues of algorithmic fairness are not of course unfounded. Algorithms are developed through programming a set of parameters, which are necessarily founded in and shaped by the values and interests of their designers—values and interests that, inescapably, become built-in to the process (Brey & Søraker, 2009). Evidence has shown that algorithmic decisions not only counteract and expose biases, but also afford new mechanisms for introducing biases with unintended and detrimental effects (Mittelstadt et al., 2016). Algorithmic decisions have been shown to amplify biases and unfairness embedded in data in relation to sensitive features such as gender, culture and race (Shrestha & Yang, 2019). Used in policing contexts, algorithmic decision-making has the potential to “compound the crisis of unfair treatment of marginalised communities … [predictive policing] provides a front of allegedly ‘impartial’ statistical evidence, putting a neutral technology veneer on pre-existing discriminatory policing practices” (Couchman, 2019: 15). Algorithms that are trained on historical data can mean that past discrimination and stereotypes prevalent in the organization and society are reflected in their predictions (Barocas & Selbst, 2016; Grimshaw, 2020; Sweeney, 2013). The application of algorithmic decision-making, derived from historical data with already embedded biases, may undermine people’s sense that the police act impartially and in a neutral fashion.

While it may seem, then, that algorithmic decision-making tools will make police decisions fairer—because they remove the potential for human bias—this is by no means a given, and there is much to suggest that bias can be “built-in” to the process. There is evidence that these sorts of concerns have filtered in “public consciousness”; Araujo et al. (2018), for example, found that their Dutch respondents expressed concern that algorithmic decision-making may lead to misuse or cause worry, and over half of the respondents thought that technology might lead directly to unacceptable outcomes. More importantly for the current study, perhaps, it also seems that people tend at the very least to be cautious about machine-driven decision-making processes and, in many cases, seem to prefer the involvement of a human being. While a system using algorithmic technology might be fully compliant with formal regulations, it may fail to have the “social license” (Brown et al., 2019) required to be accepted by the community and stakeholders within which it operates. Without this acceptability, the decisions made and outcomes achieved may not be tolerated, particularly, we might suggest, when desired ends are not achieved. Because decisions made by algorithms are opaque, lack the involvement of an identifiable human actor and are harder to trust, there is less chance for process-based factors to mitigate the effect of outcome failure.

These issues are made yet more salient by the fact that trust is vital for the acceptance of new technology, in policing and elsewhere. People who trust the police are also more willing to accept their vulnerability in relation to police—to place their trust in police—because they expect officers to be willing and able to behave fairly and effectively (Hamm et al., 2017). Trust (and/or associated constructs such as confidence and legitimacy) appears to be an important factor shaping public support for police powers, including the police potential for, and use of, force (Bradford et al., 2017; Kyprianides et al., 2021) as well as new technologies such as Body Worn Video (Lawrence et al., 2021) and the use of “big data” (Lee & Park, 2021). Trust can act as a heuristic, providing a mental shortcut towards a decision or judgement in situations where people know little about the power or technology in question (which is often the case in policing and when AI is involved). Yet, such trust is not “free-floating”, nor does it exist entirely prior to people’s encounters—of whatever kind—with police and exposure to the judgements they make. Rather, trust is developed during experiences of policing (Oliveira et al., 2020), not least because these provide people with some information about police activity, its fairness and its effectiveness. As research on algorithmic aversion implies, being exposed to apparently effective, justified and/or appropriate AI decision-making should build trust, making people more likely to accept the use of this technology (Glikson and Woolley, 2020).

## The current study

Although police organisations are increasingly turning towards automated systems, there is as yet very little evidence on how the public will respond. We do not know whether people trust algorithmic decision-making in policing, whether trust forms a basis for judging the use of algorithms (un)acceptable or indeed whether being exposed to the use of this technology in policing makes people more or less likely to accept it. Do people trust algorithmic police decision-making and do they generally approve of algorithmic policing? It is this gap that the current paper seeks to address.

We conducted an online experiment using text-based vignettes to explore perceptions of police use of algorithmic decision-making. We manipulated three factors in the vignettes. First, we manipulated whether an operational decision was made by a human (i.e. a police officer) or an algorithm. The research outlined above would suggest that while some may view algorithmic decision-making as accurate, it is more likely that people will on average be less accepting of decisions made purely by an algorithm, which they may perceive as unable to take full account of the complex characteristics of particular situations (Longoni et al., 2019) marked by inherent uncertainty (Burton et al., 2018), lacking in intuition (Lee, 2018) and/or opaque (Glikson and Woolley, 2020). Thus, we test the following hypothesis:

- H1: Participants will view police decision-making as more trustworthy, and less biased, when the decisions are made by a human (i.e. a police officer) compared to an algorithm.

Second, we manipulated the outcome of the operational decision (i.e. a reduction in crime or no change to crime levels). Previous research suggests the public are particularly concerned about the use of algorithms resulting in “unacceptable outcomes” (Araujo et al., 2018), and introducing biases and issues of accountability (Mittelstadt et al., 2016) and, in a general sense, about their reliability (Dietvorst et al., 2015; Glikson and Woolley, 2020). Thus, we test the following hypothesis:

- H2: Participants will view police decision-making as more trustworthy, and less biased, when the outcome of the decision is successful (i.e. a reduction in crime). This will be particularly true for decisions made by an algorithm; outcome success should be relatively less important in cases where the decision is made by an officer.

Third, we manipulated the type of scenario described in the vignette, which was either (1) an individual police officer in a localised situation (i.e. a stop and search decision) or (2) an area-based decision where a senior officer has to decide whether to allocate resources to a crime hotspot. While the second scenario represents most closely the way algorithmic technology is currently used within police forces in England and Wales, it would seem important to test the acceptability of algorithmic decision-making across a range of use scenarios. However, as previous research does not clearly indicate whether public perceptions will vary depending on whether the decision impacts individuals (e.g. being stopped and searched by police) or neighbourhoods (e.g. allocation of police resources), we have no a priori hypothesis for this condition.

Fourth, we consider whether being exposed to an instance of algorithmic decision-making that was judged as fair and trustworthy was also linked to greater acceptance of police use of this new technology:

- H3: Participants who view police decision-making as more trustworthy, and less biased, will be more likely to support the police use of algorithmic technology.

At the threshold, and in the context of the experiment described below, support for police use of algorithmic technology could be triggered by trust in police decision-making developed in one (or some combination) of three ways. First, human decision-making could be seen as more trustworthy than AI decision-making, meaning people in the “human” conditions may be more generally supportive of police use of algorithmic technology. Second, successful decision-making could be seen as more trustworthy than unsuccessful decision-making, meaning people in the “success” conditions, may be more generally supportive of police use of algorithmic technology. Third, it may be that it is being exposed to successful algorithmic decision-making, specifically, that generates wider acceptance of the technology. We explore these possibilities below.

## Method

### Participants

A total of 642 residents in the UK were recruited via the online crowdsourcing platform Prolific Academic on 16 November 2020.^1^ Participants were aged between 18 and 84 years old, with the majority (55%) aged between 18 and 34 years old. Females accounted for two-thirds (68%) of the participants. Some 511 participants (80%) reported their ethnic group to be White-British, White-Irish or any other White background: 9% (57) were Asian or Asian British, 5% (29) were Black or Black British, 3% (20) were mixed and 2% (15) were other. There were no significant differences in demographics across the experimental conditions. In line with Prolific recruitment protocols, participants were paid £6.02/h (£0.88) for taking part in the study.

### Procedure

The online platform Qualtrics was used to build and host the experiment.^2^ We conducted two pilot studies with a total of 440 participants, which confirmed that participants understood the scenarios and were able to appropriately answer questions based on what they had read. The experiment then used a 2 (scenario: individual vs area) × 2 (decision-making: human vs algorithm) × 2 (outcome: successful vs unsuccessful) between-subjects design.

First, participants were randomly allocated to read one of two scenarios that described either:

- *Individual*—an incident in which a single police sergeant observes suspicious males and has to make a decision about whether or not to conduct a stop and search (adapted from Ferguson, 2017).
- *Area based*—a scenario in which a crime hotspot has been identified and a police inspector has to make a decision about whether or not to direct officers to the crime hotspot for increased proactive policing (e.g. more stop and searches), thus leaving fewer resources elsewhere.

Participants were also randomly allocated to one of two decision-making conditions in which the sergeant or inspector made the operational decision, either:

- *Human*—using their own knowledge, observations and expertise.
- *Algorithm*—using a new piece of algorithmic technology incorporating a range of data/information.

In all conditions, the sergeant/inspector makes the operational decision to act (i.e. to conduct the stop and search/allocate resources to the crime hotspot). After reading the vignette, participants were randomly allocated to read one of two outcomes:

- *Successful*—in the individual scenario, the sergeant recovers items that could be used to commit a crime after conducting the stop and search. In the area-based scenario, the allocation of resources to the crime hotspot reduced crime in that area by 16%.^3^
- *Unsuccessful*—in the individual scenario, no items were recovered from the stop and search. In the area-based scenario, the allocation of resources to the crime hotspot had no impact on crime levels.

Following the vignette, participants responded to a range of questions about the decision made by the police officer described in the vignette (i.e. whether they trusted the officer had made an effective decision, was competent and unbiased in their decision-making). Participants then responded to questions about the use of decision-making technology by the police and their knowledge of algorithms.

### Constructs and measures

Confirmatory factor analysis in the package MPlus 7.11 was used to derive and validate three latent variables that comprise our dependent variables (factor scores were obtained and saved for analysis). A robust maximum likelihood approach (MLR) was used (see Appendix Table 2 for a list of the items used, factor loadings and model fit statistics). The first factor—*trustworthy decision-making—*consisted of six items measured on a 5-point agree/disagree scale and capturing whether participants thought the officer in the vignette dealt with the situation effectively and made the appropriate decision (e.g. “I would feel confident in the decision Sergeant/Inspector McFadden made” and “The Sergeant/Inspector took the most appropriate action to the situation”).

The second factor—*fair decision-making*—consisted of three items and measured whether participants thought the officer made a fair and unbiased decision (e.g. “Sergeant/Inspector McFadden’s decision making was impartial”).

The third factor—*use of technology*—consisted of five items and measured whether participants had confidence in the police use of technology (e.g. “I feel confident that technology/algorithms are accurate in the decisions they make” and “Police use of algorithms will make it easier for the police to catch criminals”). Immediately before answering these questions, participants in the officer decision-making conditions were provided a short summary of police use of algorithms, similar to that provided in the vignettes read by participants in the algorithmic decision-making conditions.

## Results

To test H1 and H2, we conducted a series of 2 (scenario: individual vs area) × 2 (decision: human vs algorithm) × 2 (outcome: successful vs unsuccessful) between subject ANOVAs with the two latent variables (trustworthy decision-making and fair decision-making) as the dependent variables. Table 1 presents the descriptive statistics for each condition.^4^

**Table 1:** Descriptive statistics for dependent variables by condition

Scenario	Decision	Outcome	*N*	Trustworthy decision-making		Fair decision-making		Support for police use of algorithms	
				*M*	SD	*M*	SD	*M*	SD
Individual	Human	Successful	81	.379	.727	.052	.765	− .309	1.01
		Unsuccessful	81	− .136	.896	− .162	.795	− .253	.965
	Algorithm	Successful	81	.315	.690	.196	.812	.138	1.01
		Unsuccessful	80	− .118	.883	− .092	.845	.059	.968
Area based	Human	Successful	80	.414	.596	.200	.677	.081	.960
		Unsuccessful	80	− .038	.726	.049	.645	.117	.988
	Algorithm	Successful	79	− .188	.786	− .022	.746	.304	.952
		Unsuccessful	80	− .638	.886	− .223	.753	− .126	.981

### Trustworthy decision-making

First, consistent with H1, there was a significant main effect of decision-making, *F* (1, 634) = 25.64, *p* < 0.001. Participants were more trusting of decisions made by a human (*M* = 0.155, SD = 0.780) compared to decisions made using an algorithm (*M* =  − 0.156, SD = 0.879). Second, consistent with H2, there was a significant main effect of outcome, *F*(1, 634) = 56.39, *p* < 0.001 with participants granting more trust when the outcome of the decision was successful (*M* = 0.232, SD = 0.741) compared to unsuccessful *(M* =  − 0.232, SD = 0.879)*.* However, we found no significant interaction between decision-making and outcome (*F* (1, 634) = 0.116, *p* = 0.733). Across both the human and algorithm decision-making conditions, participants were more trusting of decisions with a successful outcome than an unsuccessful one.

**Fig. 1:**
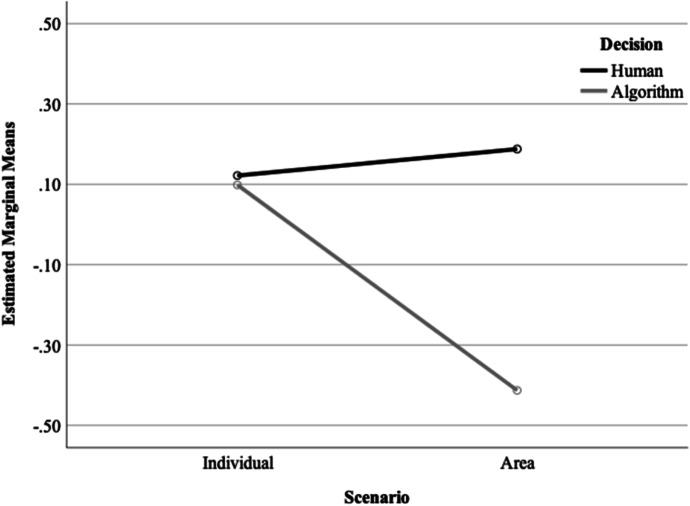
Interaction between scenario and decision-making on trustworthy decision-making

Third, we found a significant main effect of scenario, *F* (1, 634) = 13.07, *p* < 0.001. Participants were more likely to trust the decision made in the individual scenario (*M* = 0.111, SD = 0.835) compared to the area-based scenario (*M* =  − 0.112, SD = 0.841). There was a significant interaction between scenario and decision-making on trust, *F* (1, 634) = 21.95, *p* < 0.001 (see Fig. 1). In the individual scenario, the decision-making method made no difference to participants’ levels of trust: participants were equally trusting of decisions made using human experience (*M* = 0.122, SD =  0.853) and those made using an algorithm (*M* = 0.100, SD = 0.819). However, in the area-based scenario, who or what made the decision mattered. Here, participants exhibited far greater trust when the decision was made using human knowledge and experience (*M* = 0.188, SD = 0.700) than by an algorithm (*M* =  − 0.414, SD = 0.865). There were no significant interactions between the scenario and outcome (*F* (1, 634) = 0.034, *p* = 0.853) nor was there a significant three-way interaction (*F* (1, 634) = 0.105, *p* = 0.746).

### Fair decision-making

One of the biggest concerns of incorporating algorithms into operational decision-making is the potential for biases to become embedded, and that these are difficult to identify. Inconsistent with H1, we found no main effect of decision-making on the perceived fairness of the decision, *F*(1, 634) = 1.37, *p* = 0.242. Participants in the algorithm condition (*M* =  − 0.035, SD = 0.801) were just as likely to think the officer’s decision was unbiased compared to the human condition (*M* = 0.034, SD = 0.732). Yet, consistent with H2, and the findings for trustworthiness above, there was a significant main effect of outcome, *F*(1, 634) = 12.78, *p* < 0.001: decisions with a successful outcome (*M* = 1.07, SD = 0.754) were considered significantly less biased than decisions with an unsuccessful outcome (*M* =  − 1.07, SD = 0.766). Again, there was no significant interaction between decision-making and outcome, (*F* (1, 634) = 0.275, *p* = 0.600). Across both decision-making conditions, participants were more likely to perceive decisions with a successful outcome as fair.

**Fig. 2:**
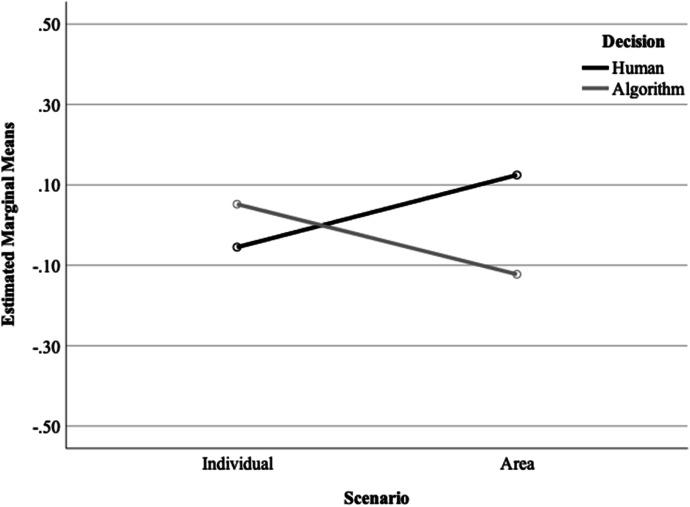
Interaction between scenario and decision-making on fair decision-making

Unlike the findings for trustworthy decision-making above, there was no significant main effect of scenario, *F*(1, 634) = 0.002, *p* = 0.966. However, there was a significant interaction between scenario and decision-making on perceptions of fair decision-making (see Fig. 2), *F*(1, 634) = 8.76, *p* = 0.003. In the individual scenario, participants were more likely to think the decision made by the algorithm was fair (M = 0.053, SD = 0.839) compared to the decision made by a human (*M* =  − 0.055, SD = 0.785). In contrast, in the area-based scenario, participants were more likely to think the decision made by a human was unbiased (*M* = 0.125, SD = 0.663), compared to the algorithm (M =  − 0.123, SD = 0.754). Again, there was no significant interaction between the scenario and outcome (*F* (1, 634) = 0.389, *p* = 0.533) and no significant three-way interaction (*F* (1, 634) = 0.011, *p* = 0.915).

### Support for police use of algorithms

The above results indicate that participants were more likely to perceive the police to have made a trustworthy, competent and unbiased decision when the decision was made by a police officer (human), and when the outcome of the decision was successful. But did the apparent trustworthiness of the decision affect support for police use of AI technology in a wider sense? As a first step towards addressing H3, we conducted the same 2 (scenario: individual vs area) × 2 (decision: human vs algorithm) × 2 (outcome: successful vs unsuccessful) between-subject ANOVA, this time with support for police use of algorithmic technology as the dependent variable (see Table 1).

**Fig. 3:**
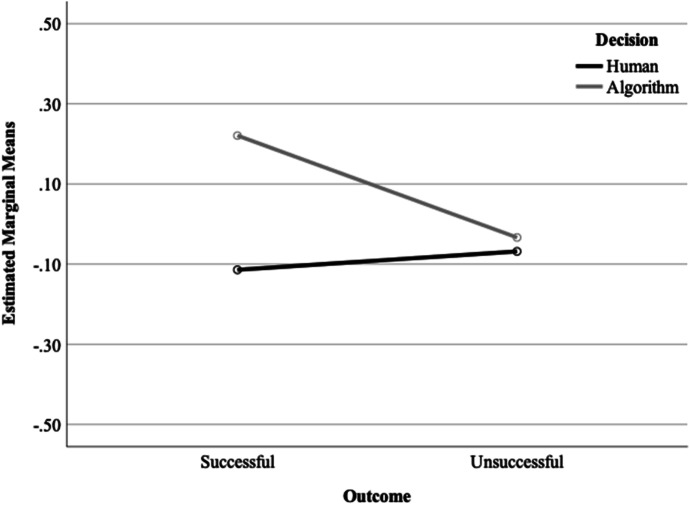
Interaction between outcome and decision-making on support for police use of algorithms

We found a significant main effect of decision-making on support for police use of algorithms, *F*(1,634) = 5.72, *p* = 0.017. Participants exposed to the algorithm vignette (*M* = 0.093, SD = 0.987) showed more subsequent support for police use of algorithms than participants exposed to the human vignette (*M* =  − 0.092, SD = 0.994). There was no significant main effect of outcome (successful vs unsuccessful); however, there was a significant interaction between decision-making and outcome at the *p* < 0.10 level, *F*(1, 634) = 3.77, *p* = 0.053—see Fig. 3. In the algorithm condition, participants were significantly more supportive of police use of technology after being exposed to a vignette with a successful outcome (*M* = 0.220, SD = 0.983) compared to an unsuccessful outcome (*M* =  − 0.034, SD = 0.976). In contrast, in the human condition, the outcome made no difference to participants’ subsequent support for police use of algorithms (successful *M* =  − 0.115, SD = 1.00; unsuccessful *M* =  − 0.070, SD = 0.991).

**Fig. 4:**
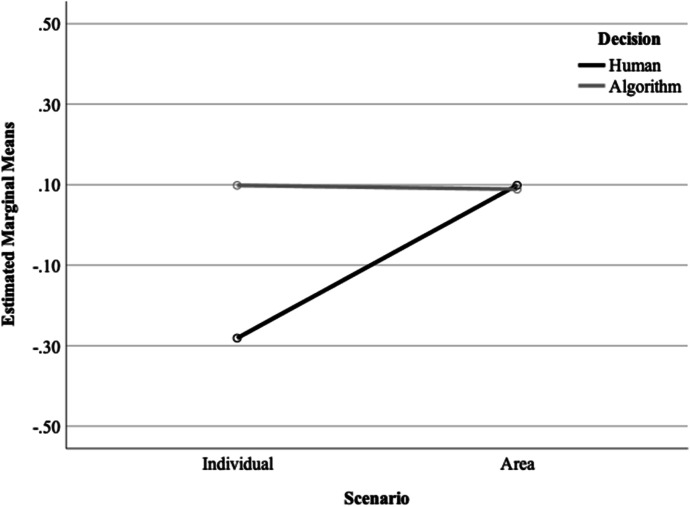
Interaction between scenario and decision-making on support for police use of algorithms

There was a main effect of scenario, *F*(1,634) = 5.74, *p* = 0.017. Participants exposed to the area scenario (*M* = 0.093, SD = 0.978) showed more support for police use of algorithms than participants exposed to the individual scenario (*M* =  − 0.092, SD = 1.00). There was a significant interaction between scenario and decision-making, *F*(1, 634) = 6.33, p = 0.012—see Fig. 4. In the algorithm condition, scenario made no difference to participants’ support for police use of technology (individual *M* = 0.099, SD = 0.989; area *M* = 0.088, SD = 0.987). In the human condition, participants were more supportive of police use of algorithms when exposed to the area-based scenario (*M* = 0.099, SD = 0.971) compared to the individual scenario (*M* =  − 0.281, SD = 0.983). There was no significant interaction between the scenario and outcome (F (1, 634) = 1.43, *p* = 0.232) and no significant three-way interaction (F (1, 634) = 1.14, p = 0.286).

**Fig. 5:**
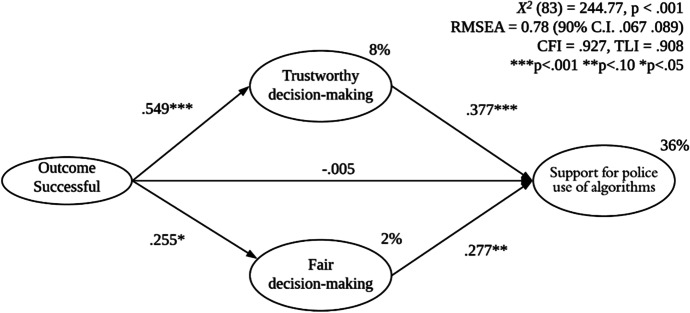
SEM predicting support for police use of algorithms

These findings indicate that exposure to a scenario in which the police used algorithmic technology to make a successful decision led participants to be more supportive of the general use of algorithms within policing. According to H3, however, this should be because exposure to a successful use case increases trustworthiness, which in turn generates support. We used structural equation modelling (SEM) in MPlus 7.11 to test the associations between perceptions of police decision-making (as trustworthy and fair) and support for the police use of algorithmic technology, and whether perceptions of trust mediated the link between scenario and support. Here, we focus only on participants in the algorithm condition since, as the analysis above suggests, they were the ones who had been exposed to a scenario from which they could make some judgement about the apparent trustworthiness of police in this area. Support for police use of algorithmic technology was regressed on trustworthiness and fairness of decision-making; decision-making was regressed on outcome condition (successful vs unsuccessful). Figure 5 presents the standardized path coefficients.

As shown in Fig. 5, both trustworthiness of decision-making (*B* = 0.377, *p* < 0.001) and fairness of decision-making (*B* = 0.277, *p* = 0.002) were significant predictors of support for police use of algorithms. In other words, respondents who felt that the police had used a particular algorithm to make a competent, effective and fair decision were more likely to support police use of new algorithmic technology. Turning to the outcome condition, as above, we find that participants exposed to a scenario where the outcome was successful were significantly more likely to grant trust (*B* = 0.549, *p* =  < 0.001) and to believe the officer had made an unbiased decision (*B* = 0.255, *p* = 0.044). Conditioning on these associations, there was no direct effect of outcome (successful vs. unsuccessful) on participants’ support for police use of technology (*B* =  − 0.005, *p* = 0.966). In other words, all of the association between outcome and support was mediated by perceptions of fair treatment and, in particular, trustworthiness.

## Discussion

Algorithms now pervade our lives. They determine the news we see, the products we buy and shape many areas of our economy and society in which new technologies and data-driven tools are being adopted in order to function more effectively and efficiently. Policing is not exempt from this process, with algorithmic decision-making and AI used more and more across multiple operational contexts. As police organisations increasingly turn towards automated systems, ethical questions arise about the police use of these new technologies—including bias and discrimination (cf. Barocas & Selbst, 2016) and the lack of transparency and accountability (cf. Citron & Pasquale, 2014)—as well as concerns about the need to maintain the public’s trust and confidence (Mackey, 2020). Yet, public reactions to the introduction of police use of algorithmic decision-making are not yet fully understood. This study sought to address this gap.

To return to the hypotheses that motivated our analysis, we found partial support for H1. Overall, people were more trusting of decisions made by a police officer compared to an algorithm. However, this effect was only present in the area-based scenario (i.e. the allocation of resources to a crime hotspot). In the individual scenario (i.e. the stop and search encounter), the decision-making method made no difference to the perceived trustworthiness of the decision. A similar pattern of results was found when looking at the perceived fairness of the decision. In the area-based scenario, participants were more likely to think the decision was fair when it was made by a police officer compared to an algorithm. In the individual scenario, there was little difference across the two decision-making conditions (although participants were slightly more likely to think the use of algorithms was fair in the individual scenario).

We also found support for H2. Across all conditions, when the outcome of the decision was successful, participants demonstrated higher levels of trust in police decision-making and perceived the decision as less biased, compared to when the decision led to an unsuccessful outcome. However, contrary to our expectations, outcome effectiveness was apparently no more (or less) important to participants in the algorithmic decision-making conditions.

Lastly, we found support for H3. Specifically, participants who were exposed to successful algorithmic decision-making expressed more support for police use of this technology, and this seemed to be because they perceived the police as being more trustworthy and fair in their decision-making (at least in comparison to those in the unsuccessful algorithmic conditions). If they are to offer their support, it is essential the public believe that any new technology introduced by the police would be effective and used appropriately. Previous research has shown that when people have trust in the police, they are more accepting of changes in the tools police use (Bradford et al., 2020). At the core of the concept of trust is a willingness to accept vulnerability in relation to the trust object (PytlikZillig & Kimbrough, 2015). What we see here may be a reflection of the fact that trust in the police is partly rooted in direct and vicarious experiences of policing (Bradford et al., 2009; Oliveira et al., 2020), which can have important implications for people’s acceptance of wider powers and policies. If people experience a particular instance of policing as trustworthy—a judgement shaped, here, by the outcome it achieves—they are more likely to support the use of powers about which, it is important to note, they are likely to know very little. Trust does, indeed, seem to be used as a heuristic.

Taken together, though, our findings suggest that the public still “prefer” decisions to be made by police officers rather than algorithms. This seems to be especially true for decisions that impact a community or neighbourhood compared to decisions that impact an individual during a one-on-one encounter, such as a stop and search. The finding that people prefer human decisions fits with theoretical perspectives suggesting that more weight is often placed on the same advice given by a human expert compared to an algorithm (Dietvorst et al., 2015). Both may be opaque, but at least with a human, one can infer trustworthy motives. When one assumes that the other is taking one’s own interests into account, one gives the decision-maker the “benefit of the doubt”.

Yet, although many of the results here reflect the “reluctant” viewpoints often associated with the acceptance and trustworthiness of algorithmic technology within the medical profession, there are also some differences. In particular, medical patients voice concerns that decision-making technology may neglect their unique characteristics, circumstances and symptoms (Longoni et al., 2019). By contrast, in our individual scenario—a face-to-face encounter—the use of algorithms was just as acceptable as the decision made by a human. Arguably, this type of situation is more similar to people’s experiences of healthcare: that decision-making technology will neglect individuals’ unique characteristics and circumstances. However, the widespread and well-documented bias and disproportionality evident in the UK criminal justice system, including in stop and search encounters (Ashby, 2020; Police Foundation, 2020), may mean the British public is particularly attuned to issues of human bias, including both overt racism and unconscious bias. Because some or indeed many people are aware that there is a current issue with disproportionality in stop and search, they may be more open to the idea of decisions being “taken over” by machines.

Indeed, public perceptions about the fairness of the police decision-making appear more nuanced than first thought, with the type of scenario being particularly important. Despite following the same set of rules and procedures every time, algorithmic technology has the potential to amplify biases and unfairness embedded in data (Mittelstadt et al., 2016; Shrestha & Yang, 2019). This could explain why participants felt that when making a decision about a community or neighbourhood (the area-based scenario), the use of an algorithm would lead to more biased decision-making. It may however be implausible to suggest the average person is sufficiently aware of algorithmic decision-making processes to draw these kinds of conclusions. Another possibility is that decisions that affect whole areas are viewed as more serious than those affecting only individual people, and this in effect raises the bar, leading people to prefer that a human actor makes the choice.

We have demonstrated here that there remains a scepticism among the public about the use of algorithmic technology, which is likely to be fuelled through the potential ethical concerns and effects of this new capability. Given this, it might seem rather paradoxical that we also found that respondents exposed to an apparently successful use case of algorithmic decision making were more likely to support police use of this power. This seems likely to reflect (a) the complexity and fuzziness of people’s opinions on the issues at hand, but also (b) how those opinions are shaped by experiences of policing (that may be trust building or undermining). Coming to the question “cold”, people preferred a human decision-maker. But having been presented with an example of apparently successful AI decision-making, those in the relevant experimental condition were more likely to support wider police use of this technology than either those exposed to an unsuccessful use case or those not exposed to an example of AI decision-making at all. Crucially, this support was forthcoming to the extent that they judged the police decision-making involved to be trustworthy.

### Limitations and future work

Some limitations of the current research must be acknowledged. First, the hypothetical nature of the scenarios described is insufficient to fully capture the nuances of how police make decisions. We used scenarios that intentionally described a situation where a police officer used solely an algorithm or solely their own knowledge and experience to make a decision in order to understand how these extreme scenarios might affect people’s views. In reality, in the UK, it is likely that the kind of scenarios presented here—a stop and search encounter and allocation of resources across a borough—would be made using a combination of decision methods. But, it is true that some police agencies in the USA have already adopted algorithmic technology (e.g. Predpol^5^) to predict when and where crime will occur. The decisions made by this technology are akin to the area-based scenario here, as Predpol identifies crime hotspots and directs resources to them. Our scenarios are also similar to those used by Ferguson (2017) when discussing issues surrounding the rise of “big data policing”.

Second, there are the typical concerns about the reliability, generalizability and validity as a result of using a non-probability convenience sample recruited from a crowdsourcing platform. While the sampling methodology used is common in the study of public attitudes towards the police (e.g. Gerber & Jackson, 2017), the results are not representative of the general population. Additionally, by virtue of the nature of the research, experimental conditions and fictional vignette scenarios cannot fully replicate real instances of police decision-making, as influential factors relating to the complexity of the decision were not fully described here. Future investigation should explore these topics from more robust methodological perspectives that use stronger manipulations or which are based on real-world interventions. For example, participants could be more exposed to police decision-making or activity via the use of CGI or virtual reality technology (Vasser & Aru, 2020) or deliberative polling methods could be used to create greater space for discussion of the inputs, risks, rewards and consequences of particular policy developments in this area.

Finally, we recommend researchers delve further into the idea that the context of the decision-making process by algorithmic technology is important. Why do the public perceive algorithmic decision-making to be less trustworthy when the decision is for a whole neighbourhood or community? Examining more nuanced applications of algorithmic technology could better elucidate the particular situations where these tools could be incorporated into operational police decisions, while gaining the support and acceptance of a currently rather skeptical public.

## Conclusion

The growth of AI, data-driven policing and algorithmic technology all provide potential ethical challenges to policing, and the proliferation of disinformation and massive growth in the use of social media are providing new opportunities to question how policing is done and at speed (Mackey, 2020). Policing methods that incorporate such technology need to be transparent and used sensitively—certainly initially only in very specific situations if the public are to be supportive of such measures. There is a clear need to maintain the public’s trust in using data for decision-making, and our results suggest that the police still have some way to go to bring the public fully on board and gain their acceptance.

A key issue here may be that of accountability. For the police to be seen as trustworthy, there must be a clear and transparent chain of command and a decision-making process that can be audited. But, identifying the human subjectivity embedded in algorithmic decision-making processes is difficult, with underlying values remaining obscured until a problematic case arises (Mittelstadt et al., 2016). Police leaders may struggle to explain what is going on “inside the box” (Mackey, 2020), not least because when harms are caused by algorithmic decisions, it can be difficult to locate the reasons due to the complex decision-making structures, hundreds of rules and probabilistic reasoning involved. In contrast to human decision-making, where an individual can usually articulate their decision-making process when required, the rationale of an algorithm is often incomprehensible to humans, making the fairness and accountability of decisions difficult to challenge (Mittelstadt et al., 2016; Vestby & Vestby, 2021). These technologies, and the opaque manner in which they are deployed, raise concerns that they may have unintended consequences and operate outside the scope of traditional oversight and public accountability mechanisms (Binns et al., 2018; Brown et al., 2019). Looking forward, it is important that the police think carefully about the situations in which they adopt algorithmic technology and follow a clear and transparent methodology that can be open to scrutiny when required. Equally, more work still needs to be done to understand public reluctance to fully accepting this transition to technology-driven decision-making.

## Supplementary Information

Below is the link to the electronic supplementary material.

Supplementary file1 (DOCX 18 KB)

## Appendix

**Table 2:** Factor loadings and model fit for confirmatory factor analysis

	Factor loadings
Trustworthy decision-making	
Sergeant/Inspector McFadden dealt with the situation effectively	0.858
I would feel confident in the decision Sergeant/Inspector McFadden made	0.858
Sergeant/Inspector McFadden demonstrated competency	0.847
The Sergeant/Inspector took the most appropriate action to the situation	0.784
Sergeant/Inspector McFadden’s actions will have helped prevent crime	0.729
The Sergeant/Inspector took all necessary information into consideration when making the decision	0.723
Fair decision-making	
Sergeant/Inspector McFadden’s decision making was impartial	0.793
Sergeant/Inspector McFadden made an unbiased decision	0.707
Sergeant/Inspector McFadden made the decision based on facts	0.690
Support for police use of algorithms	
I would feel confident if the police used technology/algorithmic tools to make operational police decisions (such as stop and search)	0.873
I feel confident that technology/algorithms are accurate in the decisions they make	0.854
Police use of algorithms makes me feel safer	0.759
The police are justified to use technology to make decisions previously made by officers in relation to operational policing	0.737
Police use of algorithms will make it easier for the police to catch criminals	0.691

Fit indices *χ*^2^(72) = 180.35, *p* < .001; RMSEA = 0.048 [.040, .057]; CFI = 0.973; TLI = 0.966

## Footnotes

1 Prolific Academic is similar to other crowdsourcing platforms such as Mechanical Turk but has a larger, more diverse pool of UK participants.

2 All materials used in this study are provided in a supplementary appendix (available here https://osf.io/dnhq8/).

3 Braga and Weisburd (2020) recently conducted meta-analyses of 53 hotspot studies and found hot spots policing generated on average a 16% (*d* = .24) reduction in crime. Therefore, this is the percentage reduction used in this study.

4 The datasets generated during and/or analysed during the current study are available in the OSF repository, available here https://osf.io/dnhq8/

5 https://www.predpol.com/
