# Supplementary material for: Artificial fairness? Trust in algorithmic police decision-making
Source: J Exp Criminol. 2021 Sep 12;19(1):165–89. doi: 10.1007/s11292-021-09484-9 (PMC8435155; doi:10.1007/s11292-021-09484-9)
Supplement: Supplementary file 1 — Supplementary file1 (DOCX 18 KB) [file 11292_2021_9484_MOESM1_ESM.docx]

**Supplementary Material**

**Scenario 1A – Individual – Human – Successful**

Sergeant McFadden is 39 years old and has worked for the police for 15 years, for many of which he has worked in a local neighbourhood team. One afternoon, Sergeant McFadden observed three men outside a jeweller. He did not know the men and had no prior reason to suspect them.

He watched from his patrol car as the men walked past the jewellers. Two of the men then broke off and made another pass in front of the shop before circling back to the third man. This pattern of walking past the shop was repeated twice more.

At that moment, Sergeant McFadden had a decision to make – should he stop and search the men to find out whether they were ‘going equipped’ to commit a robbery?

Following police guidelines, before a stop and search can be conducted, an officer must have ‘reasonable suspicion’. Reasonable suspicion is defined as “specific and articulable facts that taken with rational inferences of those facts warrant the belief that criminal activity is afoot”. Officers need to ensure the grounds for suspicion is based on facts, information, and/or intelligence.

Reasonable suspicion cannot be based on personal factors alone or stereotyping certain groups of people without reliable supporting intelligence or information or some specific behaviour by the person concerned.

Sergeant McFadden thought the men were up to no good and believed they were casing the jewellers in order to rob it. Solely on the basis of his observations and his knowledge of the local crime problems, he became increasingly suspicious. His years of experience led him to believe he had reasonable suspicion to execute a legal stop and search.

**Based solely on his experience and observations**, Sergeant McFadden decides to conduct a stop and search of all three males.

**The searches were a success** as Sergeant McFadden found a knives, crowbars, gloves and balaclavas concealed on the males. The males were all arrested on suspicion of going equipped to commit a crime and possession of a weapon. **A crime was prevented from taking place.**

**Scenario 2 – Individual – Human – Unsuccessful**

Sergeant McFadden is 39 years old and has worked for the police for 15 years, for many of which he has worked in a local neighbourhood team. One afternoon, Sergeant McFadden observed three men outside a jeweller. He did not know the men and had no prior reason to suspect them.

He watched from his patrol car as the men walked past the jewellers. Two of the men then broke off and made another pass in front of the shop before circling back to the third man. This pattern of walking past the shop was repeated twice more.

At that moment, Sergeant McFadden had a decision to make – should he stop and search the men to find out whether they were ‘going equipped’ to commit a robbery?

Following police guidelines, before a stop and search can be conducted, an officer must have ‘reasonable suspicion’. Reasonable suspicion is defined as “specific and articulable facts that taken with rational inferences of those facts warrant the belief that criminal activity is afoot”. Officers need to ensure the grounds for suspicion is based on facts, information, and/or intelligence.

Reasonable suspicion cannot be based on personal factors alone or stereotyping certain groups of people without reliable supporting intelligence or information or some specific behaviour by the person concerned.

Sergeant McFadden thought the men were up to no good and believed they were casing the jewellers in order to rob it. Solely on the basis of his observations and his knowledge of the local crime problems, he became increasingly suspicious. His years of experience led him to believe he had reasonable suspicion to execute a legal stop and search.

**Based solely on his experience and observations**, Sergeant McFadden decides to conduct a stop and search of all three males.

**The searches were unsuccessful** as Sergeant McFadden did not find anything suspicious on the males.

**Scenario 3 – Individual – Algorithmic – Successful**

Sergeant McFadden is 39 years old and has worked for the police for 15 years, for many of which he has worked in a local neighbourhood team. One afternoon, Sergeant McFadden observed three men outside a jeweller. He did not know the men and had no prior reason to suspect them.

He watched from his patrol car as the men walked past the jewellers. Two of the men then broke off and made another pass in front of the shop before circling back to the third man. This pattern of walking past the shop was repeated twice more.

At that moment, Sergeant McFadden had a decision to make – should he stop and search the men to find out whether they were ‘going equipped’ to commit a robbery?

Following police guidelines, before a stop and search can be conducted, an officer must have ‘reasonable suspicion’. Reasonable suspicion is defined as “specific and articulable facts that taken with rational inferences of those facts warrant the belief that criminal activity is afoot”. Officers need to ensure the grounds for suspicion is based on facts, information, and/or intelligence.

Reasonable suspicion cannot be based on personal factors alone or stereotyping certain groups of people without reliable supporting intelligence or information or some specific behaviour by the person concerned.

The police have recently started utilizing computer software which uses algorithms and artificial intelligence (AI) to tell officers who should be stopped and searched. Police policy dictates that officers can and should act on the result of the AI software, because the decision of the AI is considered enough, in and of itself, to constitute reasonable suspicion.

Following policy, Sergeant McFadden inputted the limited information he had available to him into the system (such as geolocation, descriptions, and observed behaviours). The new technology indicated that a stop and search was necessary.

**Based solely on the result from the software**, Sergeant McFadden then conducted a stop and search of all three males.

**The searches were a success** as Sergeant McFadden found a knives, crowbars, gloves, and balaclavas concealed on the males. The males were all arrested on suspicion of going equipped to commit a crime and possession of a weapon. **A crime was prevented from taking place.**

**Scenario 4 – Individual – Algorithmic – Unsuccessful**

Sergeant McFadden is 39 years old and has worked for the police for 15 years, for many of which he has worked in a local neighbourhood team. One afternoon, Sergeant McFadden observed three men outside a jeweller. He did not know the men and had no prior reason to suspect them.

He watched from his patrol car as the men walked past the jewellers. Two of the men then broke off and made another pass in front of the shop before circling back to the third man. This pattern of walking past the shop was repeated twice more.

At that moment, Sergeant McFadden had a decision to make – should he stop and search the men to find out whether they were ‘going equipped’ to commit a robbery?

Following police guidelines, before a stop and search can be conducted, an officer must have ‘reasonable suspicion’. Reasonable suspicion is defined as “specific and articulable facts that taken with rational inferences of those facts warrant the belief that criminal activity is afoot”. Officers need to ensure the grounds for suspicion is based on facts, information, and/or intelligence.

Reasonable suspicion cannot be based on personal factors alone or stereotyping certain groups of people without reliable supporting intelligence or information or some specific behaviour by the person concerned.

The police have recently started utilizing computer software which uses algorithms and artificial intelligence (AI) to tell officers who should be stopped and searched. Police policy dictates that officers can and should act on the result of the AI software, because the decision of the AI is considered enough, in and of itself, to constitute reasonable suspicion.

Following policy, Sergeant McFadden inputted the limited information he had available to him into the system (such as geolocation, descriptions, and observed behaviours). The new technology indicated that a stop and search was necessary.

**Based solely on the result from the software**, Sergeant McFadden then conducted a stop and search of all three males.

**The searches were unsuccessful** as Sergeant McFadden did not find anything suspicious on the males.

**Scenario 5 – Area-based – Human – Successful**

In a particular area of a UK city it has become apparent to the local police that a distinct crime ‘hotspot’ has recently emerged.

‘Hotspots’ are small areas where crime is significantly higher than elsewhere. Research evidence and practical experience suggests that conducting focused police activity in such areas – increasing police presence, using more stop and search, and engaging in other targeted interventions – can address the specific criminal behaviours involved. However, the reasons for increases in criminal activity can be complicated and hard to deal with, and focused interventions do not always produce the desired outcome.

Inspector McFadden, from the local police force, has a limited number of officers and is trying to decide where best to allocate resources. She has been policing this particular area for the past 15 years and understands the local community.

Should she direct officers to the crime hotspot and have fewer resources elsewhere?

Inspector McFadden has a decision to make. She uses her experience and recent observations to decide where to allocate extra resources.

Using her intuition, experience, and the data available to her, and after careful consideration of the likely effects of her actions, **Inspector McFadden makes the decision** and directs officers to the crime hotspot.

Two weeks later, crime statistics indicate that**crime in this hotspot area has reduced by 16%.**

**Scenario 6 – Area-based – Human – Unsuccessful**

In a particular area of a UK city it has become apparent to the local police that a distinct crime ‘hotspot’ has recently emerged.

‘Hotspots’ are small areas where crime is significantly higher than elsewhere. Research evidence and practical experience suggests that conducting focused police activity in such areas – increasing police presence, using more stop and search, and engaging in other targeted interventions – can address the specific criminal behaviours involved.

However, the reasons for increases in criminal activity can be complicated and hard to deal with, and focused interventions do not always produce the desired outcome.

Inspector McFadden, from the local police force, has a limited number of officers and is trying to decide where best to allocate resources. She has been policing this particular area for the past 15 years and understands the local community.

Should she direct officers to the crime hotspot and have fewer resources elsewhere?

Inspector McFadden has a decision to make. She uses her experience and recent observations to decide where to allocate extra resources.

Using her intuition, experience, and the data available to her, and after careful consideration of the likely effects of her actions, **Inspector McFadden makes the decision** and directs officers to the crime hotspot.

Two weeks later, crime statistics indicate that **crime levels in this hotspot area have not changed and remain high.**

**Scenario 7 – Area-based – Algorithmic – Successful**

The police have recently started utilizing computer software which uses algorithms and artificial intelligence (AI) based on intelligence and reported crime data, to indicate which areas require proactive policing (such as stop & search) and where resources need to be directed.

In a particular area of a UK city the AI has flagged a particular area is emerging as a distinct crime ‘hotspot’.

‘Hotspots’ are small areas where crime is significantly higher than elsewhere. Research evidence and practical experience suggests that conducting focused police activity in such areas – increasing police presence, using more stop and search, and engaging in other targeted interventions – can address the specific criminal behaviours involved. However, the reasons for increases in criminal activity can be complicated and hard to deal with, and focused interventions do not always produce the desired outcome.

Inspector McFadden, from the local police force, has been policing this particular area for the past 15 years and understands the local community. She has a limited number of officers and needs to decide where best to allocate resources. Police policy dictates that senior officers can and should make operational policing decisions based solely on the output from the algorithms.

As instructed, **Inspector McFadden follows the results of the algorithmic technology** which indicates that officers are directed to the crime hotspot, meaning that there are fewer resources for other parts of the area.

Two weeks later, crime statistics indicate that**crime in this hotspot area has reduced by 16%.**

**Scenario 8 – Area-based – Algorithmic – Unsuccessful**

The police have recently started utilizing computer software which uses algorithms and artificial intelligence (AI) based on intelligence and reported crime data, to indicate which areas require proactive policing (such as stop & search) and where resources need to be directed.

In a particular area of a UK city the AI has flagged a particular area is emerging as a distinct crime ‘hotspot’.

‘Hotspots’ are small areas where crime is significantly higher than elsewhere. Research evidence and practical experience suggests that conducting focused police activity in such areas – increasing police presence, using more stop and search, and engaging in other targeted interventions – can address the specific criminal behaviours involved. However, the reasons for increases in criminal activity can be complicated and hard to deal with, and focused interventions do not always produce the desired outcome.

Inspector McFadden, from the local police force, has been policing this particular area for the past 15 years and understands the local community. She has a limited number of officers and needs to decide where best to allocate resources. Police policy dictates that senior officers can and should make operational policing decisions based solely on the output from the algorithms.

As instructed, **Inspector McFadden follows the results of the algorithmic technology** which indicates that officers are directed to the crime hotspot, meaning that there are fewer resources for other parts of the area.

Two weeks later, crime statistics indicate that **crime levels in this hotspot area have not changed and remain high.**
